# The impact of consultants’ power dynamics on clients’ self-efficacy and managerial stress

**DOI:** 10.3389/fpsyg.2024.1515277

**Published:** 2025-01-21

**Authors:** Rotem Lachmi, Batia Ben-Hador, Yael Brender-Ilan

**Affiliations:** ^1^Management Consultant, Tel Aviv, Israel; ^2^Department of Economics and Business Management, Ariel University, Ariel, Israel

**Keywords:** management consulting, consultant-client dynamics, managerial stress, expert power, power bases, power relations

## Abstract

Power bases in management are crucial for leaders to effectively influence their teams and achieve organizational goals. Management consultants leverage various power bases, particularly Expert and Referent power, to influence client organizations and drive change. While previous studies have examined factors distinguishing successful consultants and the power they need to motivate clients, they focused solely on consultants’ perspectives. This study investigates the relationship between consultants’ power bases (principally Expert and Referent) and clients’ self-efficacy and managerial stress. The aim is to determine how consultants’ use of their power base affects clients’ experience and outcomes. One hundred clients participated in a study testing the correlation between consultants’ power bases and clients’ self-efficacy and stress management. Using moderation statistical models, a significant correlation was found between consultants’ Expert power and clients’ self-efficacy, with managerial stress moderating this relationship. Consulting success is measured by the extent to which it enhances clients’ self-efficacy, enabling them to effectively achieve their organizational goals and overcome challenges. The results show that Expert power is crucial in boosting clients’ self-efficacy, except under conditions of high managerial stress. This study contributes to the literature by highlighting a key power base and offering new insights into power dynamics in management consulting. Additionally, it provides practical benefits for enhancing consulting outcomes, influencing both client and consultant perspectives, and potentially improving the overall effectiveness of management consulting engagements.

## Introduction

In the complex organizational landscape, adapting through structured processes often involves collaborating with external management consultants, who offer broader insights but face challenges from organizational, political, and interpersonal dynamics (Fincham, 1999). The field of management consulting is continuously evolving in its professional and research aspects (Donelson et al., 2020). Analyzing power dynamics through Expert and Referent power bases^1^, it was found that Expert power is more influential than the Referent power base (Lachmi et al., 2024).

As more and more factors influence the consulting process, the question of what the most significant factor is in creating a successful consulting process becomes increasingly relevant. Numerous studies have focused on examining the quality of the consultant-client relationship (Chalutz Ben-Gal and Tzafrir, 2011; Sturdy and Wright, 2011; Clark and Fincham, 2002; Alvesson and Robertson, 2006; Nikolova and Devinney, 2012; Werr and Styhre, 2003), yet this question has not been fully addressed. While examining the relationship is a relatively abstract concept, the discourse emerging from the analysis of most of the research literature on management consulting emphasizes power, knowledge, and identity (Mosonyi et al., 2020). However, the ideas in the scholarship on management consulting often consider these aspects separately rather than integrating them into a comprehensive understanding of power derived from knowledge or identity. According to recent research following power relations in management consulting (Lachmi et al., 2024), the focus is on the power bases held by consultants, not merely on the overall relationship, but specifically on a dominant aspect that primarily influences the relationship between the parties and the outcomes of the consulting process. The study found that the most dominant power base is knowledge, which significantly affects the success of the consulting process. However, the study examined only the consultants’ perspective through a qualitative approach, leaving out the importance of considering both sides in the consulting process, particularly the client’s perspective.

Expert power, derived from specialized knowledge and skills, and Referent power, based on interpersonal attraction and charisma, are two key sources of influence in the consultant’s toolkit (Rahim and Buntzman, 1989). By analyzing how these power bases are utilized and perceived within client-consultant interactions, we can gain valuable insights into the nature of consulting work and its impact on organizational change processes (Issac et al., 2023). This study aims to unpack how consultants’ power bases balance their roles as advisors, facilitators, and change agents while simultaneously managing the power dynamics inherent in their professional relationships. Understanding these dynamics is crucial for both practitioners and researchers in management consulting, as it sheds light on the effectiveness of some consulting approaches and their implications for successful organizational transformations.

In management consulting, a significant research gap exists regarding the sources of the consultant’s power, particularly in the areas of Expert power and Referent (Identity) power (Lachmi et al., 2024). While these power bases are considered central to the success of the consulting process, many studies have not thoroughly explored how they affect consulting outcomes (Sturdy et al., 2004). Research indicates that expertise alone may not always be sufficient for success in the consulting process, and there is limited investigation into how expertise translates into actual results. Additionally, identity power, related to the consultant’s perceived image and reputation, affects the client’s performance; however, there is a lack of deep understanding of how identity power influences the client’s ability to implement recommendations and changes (Kipping and Clark, 2012). However, it is important to note that in this study we examine the perception of the consultant’s expertise and not the actual expertise level. Another gap relates to the impact of the consultant’s power base on the quality of the relationship between the consultant and the client (Fincham, 1999). As a result, there arose a tangible need to examine the issue of power through a quantitative study to understand the connection and correlation between factors influencing the clients’ success, such as stress and self-efficacy, regarding the main power bases—knowledge and identity. The question remains whether identity-based power has a positive correlation or knowledge-based power is more dominant. In this study consulting success is primarily determined by clients’ self-efficacy—their confidence in implementing recommendations and reaching their goals.

In the current study, a sample of 100 clients, all managers who underwent an extended consulting process in the last two years, responded to a questionnaire consisting of validated scales assessing their self-efficacy and managerial stress. Conducting this research will provide a quantitative validation of the first research question in Lachmi et al.'s (2024) study, which will either confirm or challenge the initial findings.

## Theoretical background

### Assessing the value of consulting: overcoming client’s challenges

Defining quality in institutions is generally straightforward, but it becomes challenging for services like management consulting because these services lack common measurable and comparable features (Nachum, 1999). Services without precise outcomes are even more difficult to define in terms of quality. In addition, one of the main challenges in the management consulting industry is to create high NPS (Net Promoter Score) scoring, and a high level of customer satisfaction regarding the consulting process and results (Schmuck, 2017).

This industry primarily relies on multiple success factors, due to the challenging-to-measure nature of its service outcomes. Moreover, this success requires willingness on the part of both the client and the consultant to create a successful process and positive change (Turner, 1982).

Identifying challenges involves differentiating between the consultant’s perspectives and those of the clients regarding the diverse factors that affect the success of the consulting process. There is limited theoretical research on how management consulting success factors are conceptualized from the client’s viewpoint (Bronnenmayer et al., 2016).

The combination of the rise of management sciences, together with the science of psychology and human relations theories, strengthens the need for consultants to assist and support managers in complex organizational settings (Donelson et al., 2020). Some researchers believe that just as a living organism faces challenges, so too do organizations have a natural system and mechanism capable of extracting them from problems and correcting inefficiencies (Badar, 2020). Still, this is not always the case, and organizations need the external help provided by external consultants (Donelson et al., 2020). In addition, management consultants are part of a powerful knowledge system, based on the ability to lead processes and organizational change (Will, 2015). Organizational consultants have realized that the transfer of new and contemporary knowledge is necessary to attract new clients, but this alone is no longer sufficient (Alvesson and Robertson, 2006). This is especially true because client-consultant relationships and outcomes are perceived as complex relationships that have not yet been adequately defined in the research literature (Dusén and Thörnberg, 2021).

Recent studies emphasize that the advancement of the consulting process largely depends on the quality of the client-consultant interaction and relationship (Badar, 2020). The more the client believes and trusts the consultant’s opinion and recommendations, the more he or she will want to advance the process (Dusén and Thörnberg, 2021). Thus, there is another element that affects the outcome of the consultation process: the client-consultant relationship and the client’s trust in the consultant. However, there is still a lack of knowledge about what affects this trust (Dirani et al., 2020). In the organizational consulting process, there are unconscious factors—in both the consultant and the client—driving and influencing each other’s perceptions and opinions (Stewart and Gapp, 2017). We posit that there is a need to understand the outcomes of the consulting process and its benefit to the organization – a problematic issue (Adizes et al., 2017). The definition of quality in most organizations is not very clear, nor is it easy to implement. As mentioned, sub-categories in management consulting have evolved from the same issue (Schmuck, 2017). Moreover, it is often difficult to quantify the results of the consulting process, since their impact tends to emerge over time (Tripti and Mohit, 2020). In the case of services without concrete results, it is even more challenging to define quality. Management consulting is a service that helps organizations solve organizational problems and make complex decisions (Korpiun, 2020). Therefore, it is not easy to pinpoint the actual benefits and its products. Despite these difficulties, there are several techniques that measure and may predict the success of the consulting process (Tripti and Mohit, 2020).

In addition to the difficulty of measuring the actual benefits of the consulting process and its outcomes, there is another challenge: institutionalizing the profession, and defining clear roles and responsibilities (Suddaby and Royston, 2001). The main role of the consultant is to support and advise managers regarding dilemmas in the organization, using a variety of consulting methods and tools (Visscher, 2006). However, due to the lack of uniformity in the consultant’s work and the wide variety of methods, it is difficult to evaluate the desired products properly. Some consultants are perceived as more successful, while others are perceived as having inconsistent results (Tripti and Mohit, 2020). The reasons for these differences in successful consulting processes between different consultants and clients have not yet been sufficiently examined (Adizes et al., 2017).

The present study aims to assess the real impact of a consultant’s influence on a client by closely examining the consultant’s dominant power bases and their effects on the client. There are additional factors that affect the client’s success, which will be described in the next section.

### Unidentified power bases influencing consulting outcomes

Management consultants encompass a range of solutions and methods designed to offer expert guidance to executives and managers regarding the organizational environment. The primary aims include aiding in strategic development for competitive advantages and addressing managerial and production challenges. Managers seek comprehensive solutions for complex organizational management across various sectors of activity (Ibatova et al., 2018).

Assessing management-consulting services can be challenging, due to the inability to consistently quantify results and isolate the consultant’s contribution through various influencing factors (Steinburg, 1992). To address this issue, it is important to evaluate the management consulting process by examining how it benefits both the consultant and the client, while considering both qualitative and quantitative aspects (Antonchenko and Kalenskaya, 2014).

A quantitative examination may present a clear answer to organization and managers, in contrast to qualitative aspects, which tend to be more subjective. These aspects are part of the unknown and unpredictable side of management consulting results (Ibatova et al., 2018). This study will concentrate on the key factors influencing the outcomes of the consultation process, examining the impact of the consultant’s dominant power bases on the client.

### Defining the consultant’s main power bases

Many studies focusing on power bases commonly regard Expert power has the potential to foster trust and solidarity in relationships. On the other hand, Referent power tends to have an emotional effect (Sahadev, 2005). Examining these two main power bases (Expert and Referent) has been researched previously in parallel fields. An experimental analysis was conducted to evaluate the impact of a salesperson’s Expert and Referent social power based on customer trust, attitude, and behavioral intentions. The results suggest that, in general, the knowledge and expertise power base tend to be more effective than the Identity power base, in eliciting the desired changes in customers (Busch and Wilson, 1976). However, consultants who use Referent power or the Identity power base, are more likely to share knowledge (Issac et al., 2023; Bhatt, 2001).

The power bases essentially delineate the resources at the person’s disposal regarding his ability to influence decisions. These resources form the foundation for the emotions that shape the behavioral process within the managing consulting framework. The significance and utilization of these resources play a crucial role in determining the nature of the emotions and behaviors involved (Sahadev, 2005).

#### The expert power base

Expert power is rooted in the perception of subordinates, acknowledging a superior’s possession of job experience and specialized knowledge or expertise within a particular domain (French and Raven, 1959). This variable was based on the mean score derived from six responses corresponding to the dimension in the Rahim Leadership Power Inventory (RLPI) (Rahim and Buntzman, 1989). In the field of organizational behavior and management in general, a limited number of studies have examined the Expert power base as a distinct and independent power base for empirical analysis (Sahadev, 2005).

#### The referent power base

Referent power is based on the interpersonal attraction and identification that subordinates feel towards a superior, driven by admiration or personal liking for the said superior (French and Raven, 1959). The study of Issac et al. (2023) revealed that individuals who express a strong sense of Referent power are inclined to perceive themselves as having significant influence among their colleagues. However, those with Referent power are more likely to engage in knowledge hiding (Issac et al., 2023).

Therefore, the aim of this study is to focus on both Referent and Expert power bases, and perform correlations checks in an attempt to understand whether managerial stress and self-efficacy affect success from the client’s side.

### Client’s self-efficacy integrates consulting’s outcomes

The client’s challenges in the consulting process are diverse and numerous, starting with the administrative pressure managers experience, and the multitasking they are required to do, which does not allow them the necessary stable cognitive space to dedicate themselves to change processes. This is often accompanied by other challenges including self-efficacy issues, and the nature of their relationship with their consultant. Measuring consulting success through clients’ self-efficacy provides a robust and meaningful approach to evaluating intervention effectiveness beyond traditional outcome metrics. Self-efficacy, as conceptualized by Bandura’s social cognitive theory, represents an individual’s belief in their capacity to execute behaviors necessary to produce specific performance attainments (Bandura, 1997). By focusing on self-efficacy, we aimed to capture the transformative potential of consulting interventions, which extend far beyond immediate performance outcomes to fundamental psychological mechanisms of personal agency and empowerment. This approach recognizes that successful consulting should not merely produce temporary changes but should enhance clients’ intrinsic capabilities to navigate complex professional challenges independently. The measurement of self-efficacy offers multiple methodological advantages in consulting research, providing both quantitative and qualitative insights into intervention impact. Empirical studies have consistently demonstrated that increased self-efficacy correlates with improved performance, enhanced motivation, and greater resilience across diverse domains, including organizational psychology, leadership development, and professional coaching (Stajkovic and Luthans, 2003; Chen et al., 2001). By employing validated self-efficacy scales and mixed-method approaches, researchers can capture nuanced transformations in clients’ psychological resources, tracking not just observable outcomes but the underlying cognitive and motivational shifts that sustain long-term professional growth. This approach aligns with contemporary perspectives in positive psychology and human development, which emphasize the critical role of agentic thinking in personal and professional success. Moreover, Neault et al. (2012) found that enhancing clients’ self-efficacy leads to improved performance and achievement of set goals. Thus, the success of consulting might depends on the consultant’s ability to strengthen clients’ sense of self-efficacy. Other motifs were described as ‘unknown factors,’ referring to the mystery that often arises and is discussed in research literature regarding the outcomes of consulting processes.

### Management stress

Today, the widely embraced definition of stress revolves around the interplay between individuals and their environment. Stress is the psychological and physical state that arises when an individual’s resources are inadequate to handle the demands and pressures of a given situation (Kohler and Kamp, 1992). Consequently, stress is more likely to occur in certain situations and among specific individuals. It can hinder the attainment of goals, both at the individual and organizational levels (Michie, 2002). Work-related stress is becoming more prevalent in modern society, especially among managers and executives.

Studies on organizations have found a strong connection between workplace stress and issues such as decreased job performance, acute and chronic health ailments, and employee burnout (Ivancevich et al., 1990). Williams and Cooper (1998) developed a scale that measures stress in the workplace among managers – the PMI, as described in Appendix 3- “Categorizing the variables in the stress process”.

Managers are required to simultaneously handle numerous responsibilities in the presence of various stakeholders and employees. They must often adhere to demanding schedules and, at times, work unconventional hours to accomplish their tasks. All these factors collectively contribute to heightened administrative pressure, which can affect their overall performance and self-capability (Mühlhaus and Bouwmeester, 2016).

To drive organizational change, managers must embrace the practice of “change management,” necessitating the allocation of extra resources and a mindset conducive to having a successful consulting process. Consequently, the managerial stress they experience might affect various aspects of the consulting process’s quality (Todnemt, 2005). In this context, workload refers to the effort required for managers to fulfill their job responsibilities. If people could efficiently complete all their tasks accurately and reliably using available resources, the concept of workload would not be significant. However, for managers, often concerned with organization performance, understanding operator workload is crucial. The multitude of definitions found in the psychological literature and the increasing number of identified causes, consequences, and symptoms underscore the complexity of this concept (Hart, 2006).

PMI -The Pressure Management Indicator (PMI) is a 120-item self-report questionnaire developed from the Occupational Stress Indicator (OSI). The PMI is more reliable, more comprehensive, and shorter than the OSI (Williams and Cooper, 1998). Consultants are meant to achieve various goals, one of which is providing managers with effective tools for managing their work tasks and workload, mainly reducing their managerial stress levels. This situation can be likened to a chicken-and-egg scenario: managers often face high workloads and stress, making them less available to engage in the consulting process. However, the consulting process itself aims to assist them in managing their workload efficiently, reducing stress, and fostering positive management practices (Todnemt, 2005).

### Clients’ self-efficacy as a key factor in achieving consulting goals

Studies on clients’ self-efficacy reinforce the idea that this quality is the main source of management success, task performance, and drive for change (Stajkovic and Luthans, 2003). In addition, self-efficacy is crucial to receiving and sharing complex knowledge (Endres et al., 2007). The effectiveness of consulting is largely assessed by clients’ self-efficacy, reflecting their confidence in executing recommendations and achieving their objectives (Bandura and Wessels, 1997). Self-efficacy is defined as “beliefs in one’s capabilities to mobilize the motivation, cognitive resources, and courses of action needed to meet given situational demands” (Wood and Bandura, 1989, p. 408), in the realm of research. It has been observed that managers who possess strong self-efficacy expectations exhibit the capacity to face high-risk procedures, while maintaining successful work performance effectively. Several studies have also demonstrated that one of the critical determinants of executive effectiveness, often referred to as the “active ingredients,” is closely associated with self-efficacy. Managers’ ability to believe in their capabilities can yield profound outcomes, not only in their overall life but notably in their professional endeavors (de Haan et al., 2013).

Amidst the complexities surrounding the consultant’s influence on the client, and the various factors affecting the success of the management consulting process, this study aims to bridge the gap between the consultant’s primary bases of power and an understanding of their impact on the client’s success; which is measured as enhancing clients’ self-efficacy as explained above.

In the upcoming study, the aim is to delve into the impact of the consultant’s power bases and their influence on the client’s success, considering both managerial stress (PMI) and self-efficacy (NGSE). This investigation seeks to ascertain the existence of a significant relationship between the initial variables identified in a previous qualitative study (Lachmi et al., 2024), and the variables specified in our current research.

Previous studies suggest that managerial stress may moderate the relationship between consultant’s knowledge-based power and client’s self-efficacy. For example, research has shown that high perceived power among managers enhances their managerial stress, thereby reducing emotional exhaustion (Liu and Zhou, 2020). Additionally, another study demonstrated that higher levels of self-efficacy are associated with lower levels of chronical stress (Rafiei et al., 2024). These findings indicate that strong self-efficacy may mitigate the negative effects of managerial stress, particularly when consultants utilize Expert power effectively. However, in high-stress environments, the positive impact of the consultant’s Expert power on the client’s self-efficacy might weaken, emphasizing the importance of addressing these variables when developing consulting strategies.

### The power bases model as a central framework

The PMI was chosen as a moderating variable, since the PMI is a critical factor affecting the manager’s general success (Anderson et al., 1977). The dependent variable self-efficacy (NGSE) was used. By focusing on self-efficacy, we aimed to capture the transformative potential of consulting interventions, which extend far beyond immediate performance outcomes to fundamental psychological mechanisms of personal agency and empowerment (Wood and Bandura, 1989). Self-efficacy theory (SET) is grounded in the empirically supported belief that a person’s perceived ability generates or facilitates action and change (Bandura et al., 2001).

In addition, the link between a successful management consulting process and high self-efficacy is based on the common criteria described in the literature. In relation to the management consulting field, to have a successful process, confidence in the client’s ability to do things differently during the change process is a must.

Bandura outlined the basic assumption that self-efficacy is a combination of thoughts, feelings, and behaviors. He also noted that self-efficacy was related to high goal setting, which increases our ability to imagine positive and successful scenarios. According to Bandura, self-efficacy is about trusting in one’s own abilities to succeed (Bandura et al., 2001). This approach focuses on the client’s capabilities, which are essential for a successful consulting process.

NGSE4- This is the new scale developed by Chen et al. (2001). Regarding the previous scale, the GSE had some issues such as low content validity. This new scale demonstrates high reliability in predicting specific self-efficacy. Building trust in client-consulting relationships is challenging, especially when the client’s levels of uncertainty and vulnerability are high (Nikolova et al., 2015).

The current study investigates the impact of the consultant’s power base (Expert vs. Referent) on the dependent variable of self-efficacy.

As emphasized in the literature, the client-consultant interaction is the most important factor in a successful consulting process (Schein, 1983). Primarily, the focus has been on the client-expert relationship (Kubr, 1996; Schein, 1987). Most studies in the management-consulting literature focus on the consultant. In other studies, the client-consultant interaction is presented as a shared learning process in which both parties contribute meaningful insights to the consulting process (Nikolova et al., 2009; Chalutz Ben-Gal and Tzafrir, 2011). As has been discussed in the previous qualitative research, three main themes were prevalent in the management field research from the last 28 years (219 reviewed articles): knowledge, identity, and power (Mosonyi et al., 2020). From the main insights of the diverse management consulting literature and our qualitative research, two independent variables were combined: “Expert power” and “Referent power” were taken from the Rahim Leader Power Inventory (RLPI). The power base theme was well defined by Rahim and Buntzman (1989): “…the ability of one party to change or control the behavior, attitudes, opinions, objectives, needs, and values of another party” (p. 545). As defined in Rahim’s research: (4) Expert power is based on subordinates’ belief that a superior has job experience and special knowledge or expertise in each area. (5) Referent power is based on subordinates’ interpersonal attraction to and identification with a superior due to their admiration or personal liking of the superior. One of the findings showed that Expert and Referent power bases were significantly correlated (Gaski, 1986; Rahim and Buntzman, 1989). As reflected by the Theoretical Model of Power, Conflict Styles, and Job Performance, significant positive correlations exist between Expert power and Referent power. In the present study, understanding the consequences and correlations of the client’s self-efficacy—as a key factor for a successful consulting process—will be enhanced by classifying the consultant’s main power base: Expert or Referent. Thus, we ask whether there is a relation between the consultant’s main power base, Expert or Referent, and the client’s level of managerial stress and self-efficacy. The main postulation is that the managerial pressure index (PMI) moderates the relationship between the consultant’s main power base and the client’s self-efficacy. Thus, we offer the following hypotheses:

*Hypothesis 1a*. Expert power will be positively related to the client self-efficacy.

*Hypothesis 1b*. Referent power will be positively related to the client self-efficacy.

*Hypothesis 1c*. The client’s PMI (pressure index) will moderate the relationship between the consultant’s main power base (Expert or Referent power) and the client’s self-efficacy.

To test these hypotheses, we used a questionnaire that was sent to 100 clients who had gone through a consulting process in the last two years. This research aims to validate the client’s perspective following the consultant’s view that came up in a previous qualitative research (Lachmi et al., 2024).

## Method

### Participants and procedure

The sample was comprised of clients who had engaged in a consulting process sometime during the last two years. The sample size after data cleaning was 100 participants. The size of the sample was determined sufficient for our study using GPower statisticsal power analysis calculator (Faul et al., 2007). The final sample included 50% women. The participant’s mean age was Mage = 42 (SDage = 11.57); approximately 25% held a BA degree, 58% held an MBA degree or higher. A total of 84% were in management positions (CEO, Line managers, VPs & Team leaders) with a mean role seniority of Mseniority = 7.7 years (SDseniority = 2.7). Over 53% of the participants had been through a managerial consulting process, while the Mconsulting duration = 7.5 years. Participants completed the PMI and NGSE scales. In the current study, to detect a medium effect size of 80% (Alpha = 0.05), it is recommended that the G*Power will need to have a sample of 90 participants (Sullivan and Feinn, 2012) (see Table 1).

**Table 1:** The characteristics of the research variables and sample statistics.

Variable	*N*	Range	S.D.
NGSE	100	21–40	4.44
Expert power	100	13–45	8.07
Referent power	100	6–25	3.27
PMI	100	16–86	10.2
Gender	100	0–1	5.4
Age	99	23–68	11.57
Role	100	1–26	2.7
Senior	97	1–27	2.7
Consultant type	100	0–3	0.82
Consulting duration	92	1–48	7.7
Consultant seniority	91	1–30	7.6
Client’s education	100	0–4	0.92

Participation in the study was voluntary. The researcher published posts in some management groups in social media such as LinkedIn and Facebook, in addition to using the researcher’s network connections. Moreover, the researcher sent out the survey link to the first qualitative population – consultants who probably have many clients to whom they could have sent an invitation to participate in the research. All the participants were located in Israel. The anonymity defined on the first page of the survey shows that participants agreed to take part in the study. Demographic information was collected in the first part of the questionnaire.

### Measures

The questionnaire was translated and administered in Hebrew (adapted for the research population). As described in Brislin’s (1986) An adaptation of Brislin’s Translation Model For Cross-cultural Research, we used the backward-forward translation method for each item in the questionnaire. The questionnaire was aggregated out of four validated scales.

Managerial stress—Pressure Managerial Index (PMI). The first part related to the PMI variable (Appendix 1 – Items from the PMI validated questionnaire), based on Williams and Cooper (1998) “Measuring occupational stress—The development of the Pressure Management Indicator”. The PMI Questionnaire is a 5-point Likert scale ranging from 1 = strongly disagree to 5 = strongly agree. PMI Cronbach’s alpha was 0.90.

Power Bases – Expert or Referent power of the consultant (3rd party reporting). This validated questionnaire was based on Rahim’s (1988) RLPI- Rahim Leader Power Inventory (which was based on French and Raven, 1959, and appeared in Acosta’s article from 2020). The questions which considered other power bases were removed from the questionnaire (see Appendix 2 – Rahim, 1988).

The Expert power questionnaire was composed of 10 items (Cronbach’s alpha =0.881) (which was found to be higher than Rahim’s RLPI’s Cronbach’s Alpha, Rahim and Buntzman, 1989); the Referent power questionnaire included 5 items (Cronbach’s alpha =0.71). The questionnaire was based on a 5-point Likert scale ranging from1 = strongly disagree to 5 = strongly agree.

#### Client self-efficacy (NGSE)

This index is based on a validated questionnaire that includes all the items (*N* = 8) used in the study of Chen et al. (2001, p. 62, Cronbach’s alpha =0.88; originally used in Wood and Bandura, 1989, p. 408, Cronbach’s alpha =0.85 – Appendix 3 – NGSE). The questionnaire is based on a 5-point Likert scale ranging from 1 = strongly disagree to 5 = strongly agree (see Table 2).

**Table 2:** Number of items, means, standard deviations, and Cronbach’s alpha of NGSE, expert power, referent power & PMI indexes.

Scales	Source	N. of items	Mean	SD	Cronbach a
PMI	Williams and Cooper (1998)	14	52.6	10.2	0.91
Expert power	Rahim (1988) (RLPI)	10	32.68	8.07	0.881
Referent power	Rahim (1988) (RLPI)	5	19.57	3.27	0.71
NGSE	Chen et al. (2001)	8	34.56	4.43	0.87

Client seniority, duration of consultation process and consultant’s seniority were all measured separately using a single-item open text question.

Type of consultation process the client has been through. This variable was measured using one item: “What types of consulting have you experienced?” Five different options emerged: organizational consulting, managerial consulting, coaching, business consulting, and others.

#### Control variables

The statistical analysis was controlled for respondents’ age, gender, seniority, and education to examine whether they account for some of the variance in explaining the research model.

## Results

### Preliminary analyses

Descriptive statistics and inter-correlations are shown in Table 3.

**Table 3:** Descriptive statistics and inter-correlations.

	Mean	SD	1	2	3	4	5	6	7
1. NGSE	34.56	4.43							
2. Expert power	32.68	8.07	0.556**						
3. Referent power	19.57	3.27	0.065	0.322**					
4. PMI	52.6	10.2	0.430**	0.526**	0.136				
5. Gender	2.23	5.4	0.081	−0.083	−0.107	−0.021			
6. Client’s Role	3.67	2.72	0.076	0.012	0.851	0.135	0.689**		
7. Consultant type	2.3	0.82	−0.079	0.103	0.232	0.091	−0.211*	−0.90	
8. Client’s Education	1.56	0.925	0.186	0.146	0.030	0.055	0.265**	0.178	0.073

*N* = 100. *p < 0.05, **p < 0.01, ***p < 0.001.

Before testing the hypotheses, a confirmatory factor analysis using SPSS was conducted to test the discriminant validity of the main factors. Descriptive statistics and inter-correlation with Pearson correlation were used to examine the connection between the research variables, as shown in Table 3. Significant large correlations were found between NGSE & Expert power (0.556**); Expert power & Referent power (0.322**); Expert power & PMI (0.526**); Client’s Gender & Client’s Role (0.689**); and Client’s Gender & Client’s Education (0.265**).

In addition, we conducted a manipulation check using ANOVA. An analysis of variance (ANOVA) revealed a significant difference between the independent variable –Expert power and the two dependent variables: PMI (df = 29, F [5,083] = 2.348, *p* < 0.01) and NGSE (df = 29, F [1,131] = 3.343, *p* < 0.001).

### Main analysis and hypotheses testing

Our current research model and hypotheses were tested using Hayes and Agler’s (2014) procedure to test the regression, mediation, and moderated mediation of the conceptual model. We used SPSS Macro Process 3.2, with Model 1 to test the moderation hypotheses.

Hypothesis 1a proposed that Expert power will be positively related with the client’s self-efficacy (NGSE). Hypothesis 1a was supported. Specifically, the more the client perceives that the consultant utilizes his/her Expert power the more the client will tend to exhibit higher self-efficacy levels (t = 0.31, *p* < 0.000). Hypothesis 1b proposed that Referent power will be positively related to the client’s self-efficacy (NGSE). This hypothesis was not confirmed (*t* = 0.09, *p* = 0.52).

Hypothesis 1c proposed that PMI will moderate the relationship between the consultant’s primary power base (Expert or Referent power) and the client’s self-efficacy. Specifically, when the PMI is higher, it is anticipated that this will result in a less pronounced correlation between the consultant’s primary power base and the client’s self-efficacy.

**Figure 1:**
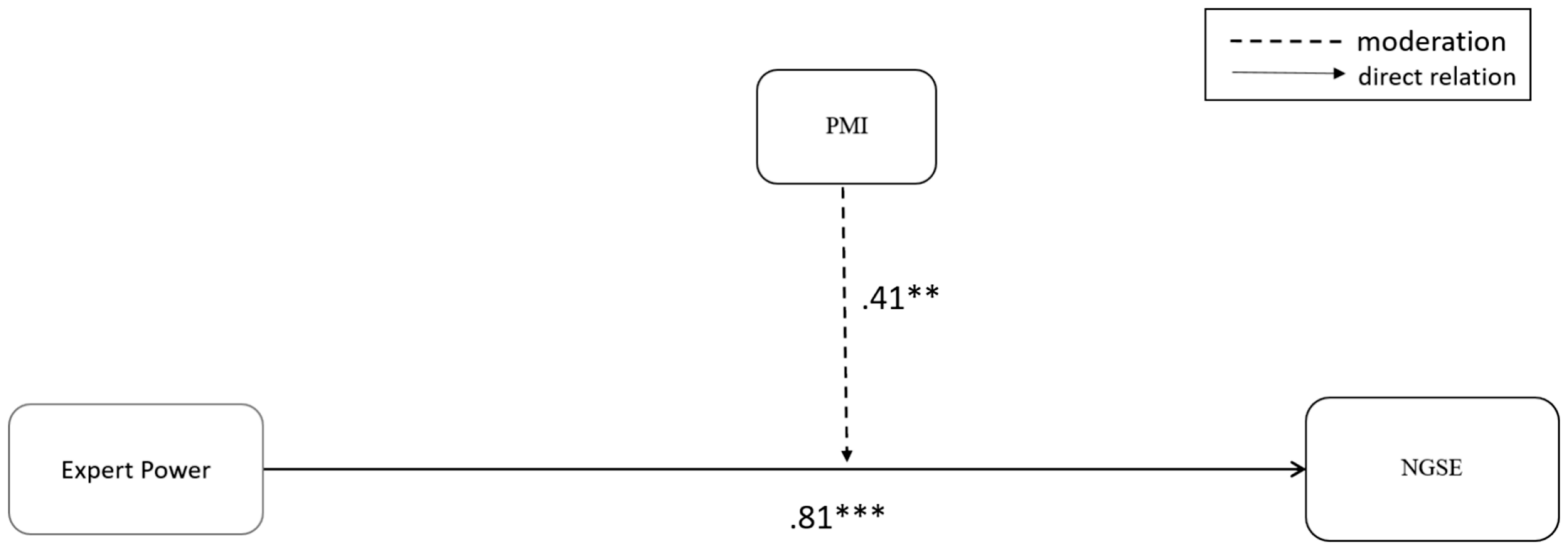
The moderation effect of PMI on the relationship of expert power and the client’s self-efficacy (NGSE).

**Figure 2:**
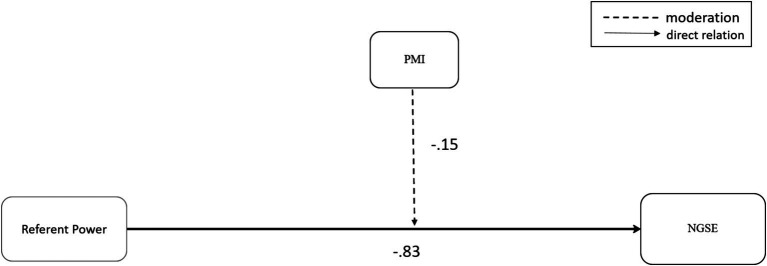
The moderation effect of PMI on the relationship of referent power and the client’s self-efficacy (NGSE).

Figures 1, 2 demonstrate the moderation results of the two power bases, Expert power and Referent power. A moderation effect was found for the relationship of the consultant’s Expert power and the client’s self-efficacy. In contrast, for Referent power, no such moderation was shown and the direct relation was not significant (see Figures 1, 2).

Comparing those results to French and Raven’s study (1959, Appendix 3 – Theoretical Model of Power, Conflict, and Job Performance), it is clearly understood from their research that these two power bases are the main ones: Referent and Expert power. In contrast to their study, the findings of this research point to Expert power as the most significant source of a consultant’s power and influence.

**Figure 3:**
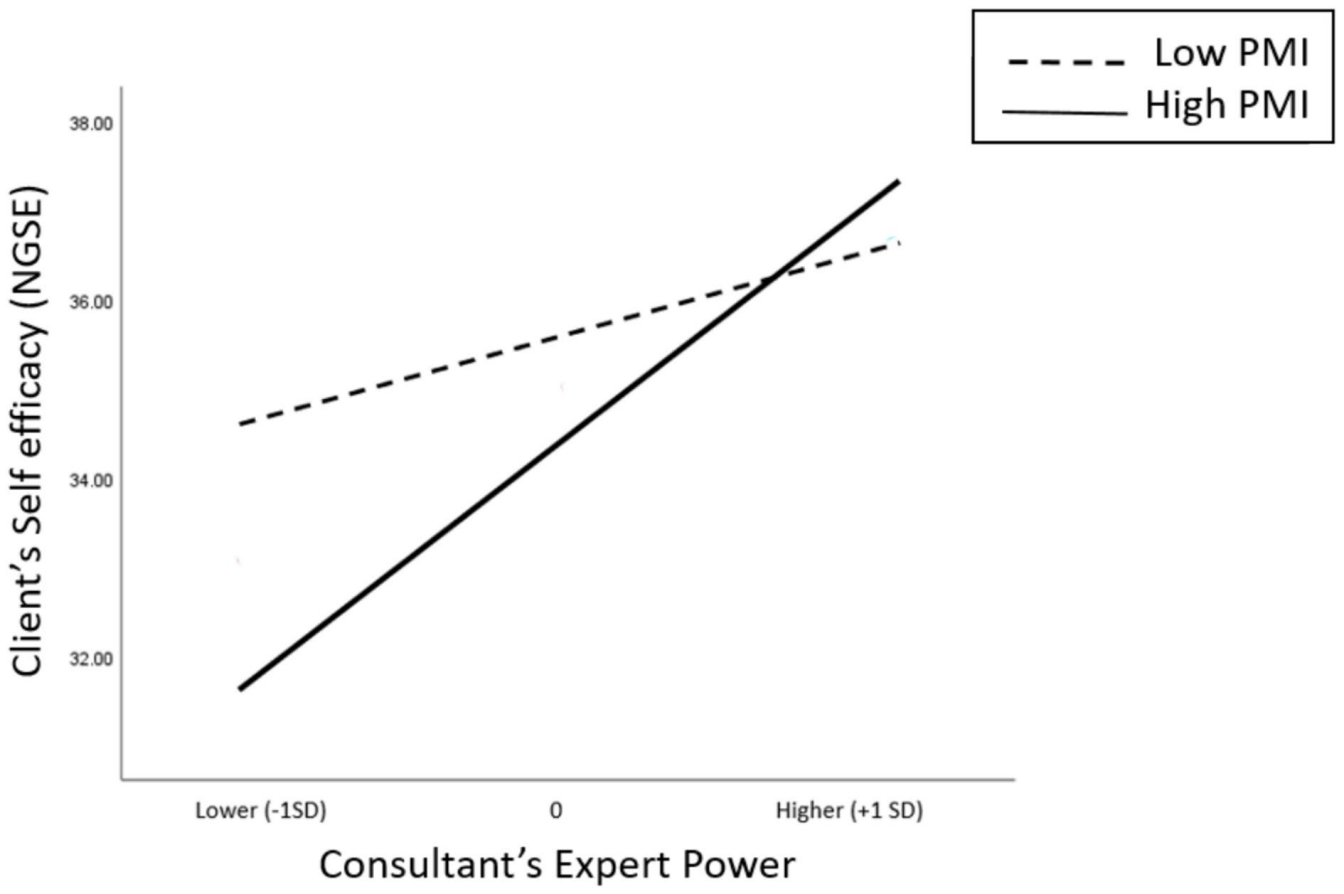
The interaction between the client’s self-efficacy (NGSE) and the consultant’s expert power base, on levels of client’s management stress (PMI).

We examined the moderation mediation hypothesis for PMI on Expert power and NGSE. A significant interaction relationship was found (R^2^-chng = 0.043, *F* = 6.58, *p* = 0.0119) and is depicted in Figure 3. The conditional effects of PMI on NGSE at values of consultant’s Expert power are shown in Table 4.

**Table 4:** Conditional effects of PMI on NGSE at values of consultant’s expert power.

National pride	Effect	*p*	SE	95% LLCI	95% ULCI
Low level (−1 SD)	0.352	<0.001	0.065	0.2224	0.4820
High level (+1 SD)	0.139	0.04	0.068	0.0040	0.2734

LLCI = Lower level confidence interval; ULCI = Upper level confidence interval.

Specifically, for consultants with high Expert power, clients’ self-efficacy level is high and similar for high and low levels of clients’ PMI. However, for consultants with low Expert power, clients’ self-efficacy level depends on their level of PMI. That is, for clients with low PMI, the self-efficacy level is higher compared to that of clients with high PMI.

## Discussion

The research results shed new light on the main power bases utilized during the management consulting process. The current research documents significant associations linking the ways in which Expert or Referent power bases used during the management consulting process affect the client’s self-efficacy. However, the previous research literature addressed knowledge, identity, and power as separate themes, rather than one unified theory related to power-relations (Mosonyi et al., 2020). The study showed a robust and positive influence regarding the consultant’s use of Expert power and the consultant-client relationship. Our findings corroborate previous qualitative results (Lachmi et al., 2024). Our study was conducted to ascertain the relative strength of the two power bases – Expert and Referent. We aimed to explore what base has more effect on the client. This study has shown significant results regarding the use of Expert power compared to insignificant results regarding the use of Referent power. A significant positive correlation was found between the consultants’ Expert power and the client’s self-efficacy, while the client’s managerial stress moderated this connection. These findings emphasize and focus on the importance of consultants’ Expert power in order to deal successfully with their clients. Hence, the current study confirmed and strengthened the findings of the previous qualitative study (Lachmi et al., 2024) and adds a unique addition to its findings, the effect of the significant correlation between the consultant’s Expert power base and the client’s self-efficacy.

Our recent discoveries have significant theoretical contributions, particularly in the realm of management consulting. Primarily, our findings add to the growing body of knowledge regarding the influence of power dynamics, emphasizing the Expert power base within the management consultant field (Buono, 2002; Sturdy et al., 2004). Furthermore, this study emphasizes the importance of knowledge as the cornerstone of management consulting (Lahti and Beyerlein, 2000; Anand et al., 2007; Mosonyi et al., 2020).

Self-efficacy among managers has been found to be one of the important factors for their success, as managers need to deal with multiple tasks and a rapidly changing environment (Richter and Schmidt, 2008). Studies focusing on the client’s self-efficacy, found it to be a main factor driving change, and increasing management performance and task management – all of which are assumed to be key factors for success in the consulting process (Clark and Fincham, 2002; Richter and Schmidt, 2008; Endres et al., 2007). Indeed, many studies thus far have emphasized different factors such as: the client’s skills, intensity of collaboration, common vision, and more (Bronnenmayer et al., 2016). A novel aspect of our research is that it highlights the client’s self-efficacy as a pivotal success factor. Lastly, our findings regarding the managerial stress factor shed new light on some barriers to the management consulting process from the client’s perspective that were not mentioned in theoretical discussions. Managerial stress was found to be a moderating variable, moderating the positive correlation between the consultant’s Expert power base and the client’s self-efficacy. The previous study highlights the consultant’s role in shaping success, emphasizing their knowledge and expertise (Expert) power base, while the current study focuses on the client’s influence (self-efficacy and managerial stress). The synthesis of the two studies reveals a clear positive linkage between the consultant’s Expert power base and the client’s self-efficacy, while the client’s managerial stress moderates this correlation. This pioneering research establishes a novel linkage between the potency of Expert power as a primary resource wielded by the consultant to enable a positive influence and the empowerment of the client’s self-efficacy for action and organizational transformation.

### Theoretical implications

Expert power is well-known in the current literature as one of the power bases first discussed in French and Raven’s power taxonomy in French and Raven (1959), and later in Rahim’s leader RLPI (Rahim and Buntzman, 1989). It has been widely used in some parallel fields such as psychology, education, and work in organizations; however, none of the previous studies considered the field of management consulting (Robyak et al., 1987; Wexley and Snell, 1987). This study suggests that, in the realm of the management consulting field, consultants’ primary power base is their knowledge and expertise – or their Expert power.

Moreover, the discussion about which factors have the largest influence on the client’s success in the management consulting process was divided by many diverse ideas, theories and criteria, none of which related to the client’s managerial stress or self-efficacy (Kipping and Clark, 2012). Furthermore, in the literature, client’s perspective received very little research attention, as most studies focused on the consultants’ abilities and conceptual forms (Bronnenmayer et al., 2016). Our study sheds new light on the subject and emphasizes the client’s self-efficacy, which is significantly correlated with the consultant’s main power base – Expert power.

### Practical implications

The current study provides important practical implications for management consultants and their clients from all over the world. Management consultants may simplify managers’ work, making them more efficient and improving their organizational environment by using up-to-date knowledge (Sturdy et al., 2004; Bronnenmayer et al., 2016). The consultant’s use of the Expert power base, and the client’s managerial stress levels and sense of self-efficacy are all critical to a successful consulting process.

The practical implications of this study affect both the consultant and the client. Up until now, the existing paradigm emphasized leadership theories, while this study focuses on consultants’ knowledge and expertise as their central and dominant source of power. For the most part, the extensive research literature stresses consultants’ personality characteristics, leadership styles, and the ways these influence the clients and the consulting process. This study changes the rules of the game and focuses, instead, on consultants’ knowledge. Hence, the practical implication is on professional development; exposure to a wide range of organizational and managerial complexities enriches and improves the quality of consultants.

Together with this, the clients’ awareness and alertness to their situation allows for a smart observation of the stage at which they choose to start the consulting process. Both in the literature and in practice, managers tend to start consulting processes when they experience high levels of managerial stress. According to the present study, starting the consulting process during a time of administrative burden and pressure may lead to undesirable results. This is because managerial stress was found to be a factor that moderates the influence of the consultant’s Expert power base. The clients, therefore, must be vigilant and even preventive, and start the consulting process before experiencing high levels of managerial stress. This will allow them to implement the necessary change processes recommended by the consultant.

### Limitations and future directions

Although the existing research design exhibits several commendable attributes, there are still some research limitations. The perspective of clients, who had gone through a consulting process in the last two years, was self-report and as such is susceptible to self-report study limitations (Chan, 2010). Also, these clients were exclusively from one country, whereas the consultants in the qualitative study were from several countries. We suggest a follow-up study with a global population and a sample of additional clients from several countries to strengthen the results of the current study. Moreover, further research is also essential to unravel the mechanisms through which information is conveyed by consultants. Additionally, there is a need to advance the practice of dyadic research, despite its inherent challenges and the reluctance of consultants and clients to participate in such studies. Dyadic research in the consulting context demands a high degree of sensitivity and often encounters resistance. Simultaneously, conducting a follow-up study that incorporates a questionnaire designed for consultants, coupled with an assessment of clients’ self-efficacy (and potentially the exploration of other relevant factors), has the potential to shed additional light on the subject and verify the findings of the current study. Another limitation that is important to mentions is that despite using a large sample, the study’s findings might not be universally applicable across all industries, cultural contexts, or consulting scenarios. Lastly, the study examines the influence of the consultant on the client only. There is certainly a mutual influence and interaction between the client and the consultant. The client also operates under the influence of hidden and visible sources of power. In the present study, the focus was on consultants’ influence on clients. Future studies that examine clients’ power bases and their influence, in addition to the mutual interaction between consultant and client, are recommended.

## Data Availability

The raw data supporting the conclusions of this article will be made available by the authors, without undue reservation.

## Footnotes

^1^Expert power—The term used in the literature to describe a source of power based on knowledge and expertise (French and Raven, 1959; Rahim and Buntzman, 1989). Referent power—The term used in the literature to describe a source of power based on identity and personal skills (French and Raven, 1959; Rahim and Buntzman, 1989).
