# Supplementary material for: The impact of consultants’ power dynamics on clients’ self-efficacy and managerial stress
Source: Front Psychol. 2025 Jan 21;15:1515277. doi: 10.3389/fpsyg.2024.1515277 (PMC11790657; doi:10.3389/fpsyg.2024.1515277)
Supplement: Supplementary file 1 [file Data_Sheet_1.docx]

Appendix 1 - PMI

| Items | |
| --- | --- |
| 1. | I feel stability in the organization |
| 2. | I feel that I have job security |
| 3. | I feel committed to the organization |
| 4. | I feel that my organizational environment is getting better |
| 5. | I feel satisfied with my work (in terms of actions and tasks) |
| 6. | I am satisfied with the way the organization is structured |
| 7. | I am satisfied with the way the organization functions |
| 8. | I am satisfied with my mindset |
| 9. | I am satisfied with my personal sense of calm |
| 10. | I feel full of vigor, energy, and vitality during the day |
| 11. | I feel pressured by the amount and complexity of the tasks I must perform |
| 12. | I get along with the people in my work environment |
| 13. | I feel the need to be appreciated for my achievements at work |
| 14. | I feel the need to succeed and achieve results at work |

| Appendix 2  RLPI - Rahim, 1988 (Referent & Expert power only) | | | |
| --- | --- | --- | --- |
| Items | | Expert power | Referent power |
| 1. | My advisor has a pleasant personality |  | V |
| 2. | I go to my consultant about work-related issues because he\she's usually right | V |  |
| 3. | My consultant knows how to find the information I need to improve my performance | V |  |
| 4. | My consultant has the practical knowledge to assist me in doing complicated work | V |  |
| 5. | From my consultant’s point of view, it seems very logical to him to decide for me which organizational tasks I will do and when | V |  |
| 6. | My consultant has a specific specialization in his field (for example "consultant for hi-tech organizations\ strategy consultant\ financial consultant and more) | V |  |
| 7. | My consultant does not have the knowledge and expertise to improve my performance in the organization | V |  |
| 8. | I do not want to be identified with my consultant |  | V |
| 9. | My consultant knows how to provide me with relevant information that will help me in performing my work | V |  |
| 10. | I prefer to do what my consultant suggests because he is more expert than me | V |  |
| 11. | My consultant has access to the information I need to do my job better | V |  |
| 12. | 12. My consultant knows how to treat everyone fairly |  | V |
| 13. | My consultant can provide information that will help the proper operation of my department / organization | V |  |
| 14. | I like my consultant’s personal qualities |  | V |
| 15. | My consultant is not the type of person I like to work with |  | V |

Appendix 3

New general Self-Efficacy Scale (NGSE)

|  | Items |
| --- | --- |
| 1. | I will be able to achieve most of my goals that I have set for myself |
| 2. | When facing difficult tasks, I am certain that I will accomplish them. |
| 3. | In general, I think that I can Obtain outcomes that are important to me. |
| 4. | I believe I can success at most any endeavor to which I set my mind. |
| 5. | I will be able to successfully overcome many challenges. |
| 6. | I am confident that I can perform effectively on many different tasks. |
| 7. | Compared to other people, I can do most tasks very well. |
| 8. | Even when things are tough, I can perform quite well. |
